# 5,11,17,23-Tetra­kis(chloro­meth­yl)-25,26,27,28-tetra­propoxycalix[4]arene

**DOI:** 10.1107/S160053681100660X

**Published:** 2011-02-26

**Authors:** Felix Kutter, Matthias H. Düker, Matthias Zeller, Vladimir A. Azov

**Affiliations:** a University of Bremen, Department of Chemistry, Leobener Strasse, NW 2C, D-28359 Bremen, Germany; b Youngstown State University, One University Plaza, Youngstown, OH 44555-3663, USA

## Abstract

The title calix[4]arene, C_44_H_52_Cl_4_O_4_, displays the 1,3-alternate conformation with crystallographically imposed twofold symmetry. Four phenolic rings of the calixarene backbone are tilted into the calix cavity, making dihedral angles of 77.42 (2) and 77.71 (2)° with the plane of the four bridging methyl­ene C atoms. Pairs of opposite aromatic rings make dihedral angles of 25.16 (3) and 24.58 (4)° with each other. In the crystal, the calixarene mol­ecules pack with the formation of infinite columns along the *b* axis. The crystal packing shows a network of C—H⋯Cl contacts, which can be considered as non-classical hydrogen bonds.

## Related literature

For calixarene derivatives and their applications, see: Gutsche (2008 ▶); Ikeda & Shinkai (1997 ▶). For the use of calixarenes in crystal engineering, see: Dalgrano *et al.* (2007 ▶). For the previous synthesis of the title compound, see: Ikeda & Shinkai (1994*a* ▶). For its application in the formation of nanotubes, see: Ikeda & Shinkai (1994*b* ▶). For reviews on weak non-classical hydrogen bonding, see: Desiraju & Steiner (1999 ▶); Steiner (2002 ▶); Desiraju (2005 ▶).
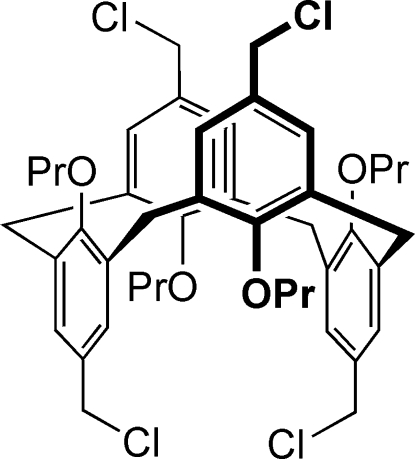


## Experimental

### 

#### Crystal data

C_44_H_52_Cl_4_O_4_
*M* *_r_* = 786.66
Monoclinic, 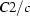

*a* = 23.104 (3) Å
*b* = 11.5871 (15) Å
*c* = 17.618 (2) Å
β = 117.655 (2)°
*V* = 4177.7 (9) Å^3^
*Z* = 4
Mo *K*α radiation
μ = 0.32 mm^−1^
*T* = 100 K
0.49 × 0.31 × 0.15 mm

#### Data collection

Bruker Kappa APEXII CCD diffractometer
Absorption correction: multi-scan (*SADABS*; Bruker, 2009 ▶) *T*_min_ = 0.658, *T*_max_ = 0.746
15796 measured reflections
6176 independent reflections
5280 reflections with *I* > 2σ(*I*)
*R*_int_ = 0.019

#### Refinement

*R*[*F* ^2^ > 2σ(*F* ^2^)] = 0.045
*wR*(*F* ^2^) = 0.119
*S* = 0.97
6176 reflections
235 parameters
H-atom parameters constrained
Δρ_max_ = 0.84 e Å^−3^
Δρ_min_ = −1.05 e Å^−3^

### 

Data collection: *APEX2* (Bruker, 2009 ▶); cell refinement: *SAINT* (Bruker, 2009 ▶); data reduction: *SAINT*; program(s) used to solve structure: *SIR97* (Altomare *et al.*, 1999 ▶); program(s) used to refine structure: *CRYSTALS* (Betteridge *et al.*, 2003 ▶); molecular graphics: *ORTEP-3* (Farrugia, 1997 ▶) and *Mercury* (Macrae *et al.*, 2006 ▶); software used to prepare material for publication: *CRYSTALS*, *enCIFer* (Allen *et al.*, 2004 ▶) and *publCIF* (Westrip, 2010 ▶).

## Supplementary Material

Crystal structure: contains datablocks I, global. DOI: 10.1107/S160053681100660X/rk2266sup1.cif

Structure factors: contains datablocks I. DOI: 10.1107/S160053681100660X/rk2266Isup2.hkl

Additional supplementary materials:  crystallographic information; 3D view; checkCIF report

## Figures and Tables

**Table 1:** Hydrogen-bond geometry (Å, °)

*D*—H⋯*A*	*D*—H	H⋯*A*	*D*⋯*A*	*D*—H⋯*A*
C22—H222⋯Cl25^i^	0.97	2.90	3.786 (1)	153
C23—H231⋯Cl26^ii^	0.97	2.90	3.557 (2)	127

Symmetry codes: (i) 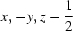; (ii) 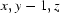.

**Fig. 1.**
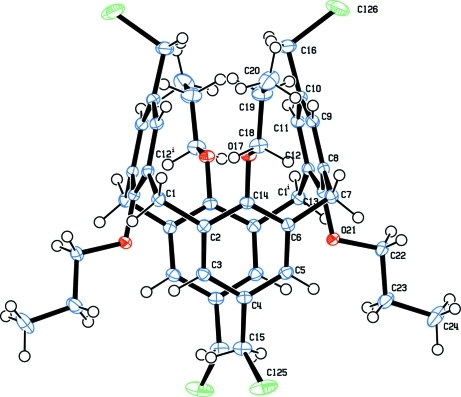
ORTEP–3 plot of the title molecule with the atom numbering scheme. Displacement ellipsoids are represented at 50% probability levels. H atoms are presented as a small spheres of arbitrary radius. Symmetry code: (i) -x+1, y, -z+3/2.

**Fig. 2.**
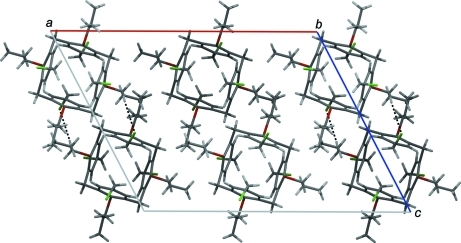
Crystal packing of the title compound viewed along the b axis into the infinite channels formed by the calixarene backbones. Short C—H···Cl contacts, interconnecting pairs of molecules along the c axis, are shown as dotted lines.

**Fig. 3.**
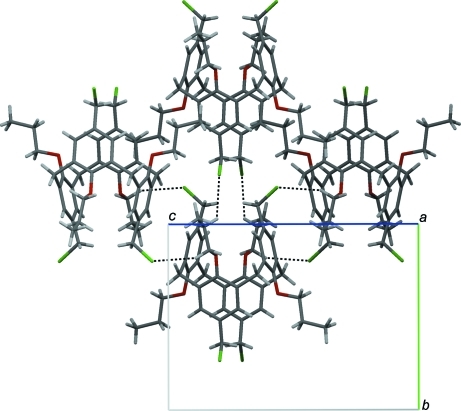
Packing of the title compound viewed along the a axis. Short C—H···Cl contacts, interconnecting pairs of molecules along the b axis (vertical) and c axis (horizontal), are shown as dotted lines.

## supplementary crystallographic information

### Comment

Calixarenes, a family of macrocyclic compounds, have shown to be superb molecular scaffolds for the construction of macromolecular and supramolecular architectures (Gutsche, 2008; Ikeda & Shinkai, 1997). Calix[4]arenes can adopt several conformations, of which the *cone* conformation is the most commonly employed one. Due to their bowl shape and ease of preparation, they are employed widely in supramolecular chemistry and crystal engineering (Dalgrano *et al.*, 2007) for preparation of species and materials suitable for molecular encapsulation. The *1,3–alternate* conformation of calix[4]arenes is much less commonly used. The title compound and its derivatives were previously synthesized (Ikeda & Shinkai, 1994*a*) to study binding of metal cations in solution, as well as for preparation of calixarene–based nanotubes (Ikeda & Shinkai, 1994*b*).

The molecule of the title compound is shown in Fig. 1. The calix[4]arene bowl adopts the *1,3–alternate* conformation around a twofold symmetry axis; for that reason, the IUPAC numbering scheme for calix[4]arenes could not be applied. All bond lengths and angles may be considered normal. Four phenolic rings are pitched into the calix cavity, as defined by the angles, which the aromatic rings make with the plane of the four bridging methylenes (C1–C7–C1^i^–C7^i^): 77.42 (2)° (ring C2–6, C14) and 77.71 (2)° (ring C8–13), respectively (symmetry code: (i) -*x*+1, *y*, -*z*+3/2). Two pairs of opposite aromatic rings show interplanar angles of 25.16 (3)° (ring C2–6, C14) and 24.58 (4)° (ring C8–13), respectively. Four propyl chains point outside the cavity and adopt an *anti* conformation for all their bonds. Four chlorine atoms are also pointing outside from the calix cavity.

Several non–classical intermolecular weak hydrogen bonds are present in the structure (Desiraju & Steiner, 1999; Steiner, 2002; Desiraju, 2005). Details of the packing interactions are given in Table 1. Molecules pack into infinite columns along the *b* axis. Two short C23–H231···Cl26^iii^ (symmetry code: (iii) *x*, *y*-1, *z*) contacts (2.90Å), parallel to the *b* axis, link molecules with each other (Fig. 2). Along the *c* axis, the molecules are interconnected side–to–side through pairs of C22–H222···Cl25^ii^ (symmetry code: (ii) *x*, -*y*, *z* - 1/2) interactions (2.90Å, Fig. 3). In both cases, hydrogen atoms of the C22–24 propyl chains serve as H–bond donors. When viewed along the *b* axis, calixarene backbones form infinite channels with a shortest distance of 8.8090 (13)Å between the two neighboring channel centers (Fig. 2).

### Experimental

A solution of 25,26,27,28–tetrapropoxycalix[4]arene (0.108 g, 0.169 mmol), paraformaldehyde (0.115 g, 3.83 mmol), glacial acetic acid (1.3 ml), and conc. H_3_PO_4_ (1.3 ml) in dioxane (5 ml) was stirred for 2 h at 353 K. After addition of conc. HCl (1.3 ml, 16.1 mmol) the solution was stirred for additional 16 h at 353 K. The mixture was concentrated under vacuum up to *ca* 3 ml, poured into ice/water (100 ml) and extracted with CH_2_Cl_2_ (3×20 ml). The combined organic phases were washed with water and brine, dried (Na_2_SO_4_), and evaporated to dryness. The resulting oil was dissolved in a small amount of CH_2_Cl_2_ and *Me*OH was slowly added. The precipitate was filtered off, washed with cold *Me*OH, dried under vacuum, and purified by column chromatography to yield 80 mg (0.102 mmol, 60%) of product as a white crystalline powder.

*R*_f_ = 0.41 (CH_2_Cl_2_/PE, 1:1). Mp: 562–565 K (CHCl_3_/heptane, decomp.); Lit: 556–558 K (Ikeda & Shinkai, 1994*a*). ^1^H NMR (200 MHz, CDCl_3_): δ 1.02 (t, *J* = 7.5 Hz, 12 H), 1.78 (tq, *J* = 7.2, 7.5 Hz, 8 H), 3.55 (s, 8 H), 3.63 (t, *J* = 7.2 Hz, 8 H), 4.43 (s, 8 H), 7.01 (s, 8 H). ^13^C NMR (50 MHz, CDCl_3_): δ 10.6, 23.8, 36.0, 46.7, 73.8, 129.8, 130.5, 133.3, 156.7. HR–MS (EI, 70 eV): *m*/*z* 784.25829 (*M*^+^, C_44_H_52_Cl_4_O_4_^+^, calcd. 784.26197).

X–ray quality crystals were grown by slow evaporation of a chloroform/heptane solution and appeared as large (up to 1–2 mm) transparent blocks.

### Refinement

All non–hydrogen atoms were refined with anisotropic displacement parameters. All H atoms were located in electron difference density maps and initially refined with soft restraints on the bond lengths and angles to regularize their geometry (C—H in the range 0.93Å–0.98Å) and *U*_iso_(H) (in the range 1.2–1.5 times *U*_eq_ of the parent atom), after which the positions were refined with riding constraints.

### Crystal data

**Table:**

C_44_H_52_Cl_4_O_4_	*F*(000) = 1664
*M**_r_* = 786.66	*D*_x_ = 1.251 Mg m^−^^3^
Monoclinic, *C*2/*c*	Melting point = 562–565 K
Hall symbol: -C 2yc	Mo *K*α radiation, λ = 0.71073 Å
*a* = 23.104 (3) Å	Cell parameters from 6719 reflections
*b* = 11.5871 (15) Å	θ = 2.6–31.2°
*c* = 17.618 (2) Å	µ = 0.32 mm^−^^1^
β = 117.655 (2)°	*T* = 100 K
*V* = 4177.7 (9) Å^3^	Plate, colourless
*Z* = 4	0.49 × 0.31 × 0.15 mm

### Data collection

**Table:**

Bruker Kappa APEXII CCD diffractometer	6176 independent reflections
Radiation source: fine–focus sealed tube	5280 reflections with *I* > 2σ(*I*)
graphite	*R*_int_ = 0.019
ω scans	θ_max_ = 31.3°, θ_min_ = 2.0°
Absorption correction: multi-scan (*SADABS*; Bruker, 2009)	*h* = −32→21
*T*_min_ = 0.658, *T*_max_ = 0.746	*k* = −16→16
15796 measured reflections	*l* = −25→24

### Refinement

**Table:**

Refinement on *F*^2^	Primary atom site location: structure-invariant direct methods
Least-squares matrix: full	Hydrogen site location: inferred from neighbouring sites
*R*[*F*^2^ > 2σ(*F*^2^)] = 0.045	H-atom parameters constrained
*wR*(*F*^2^) = 0.119	Method: Modified Sheldrick *w* = 1/[σ^2^(*F*^2^) + (0.06*P*)^2^ + 6.5*P*], where *P* = (max(*F*_o_^2^,0) + 2*F*_c_^2^)/3
*S* = 0.97	(Δ/σ)_max_ = 0.001
6176 reflections	Δρ_max_ = 0.84 e Å^−^^3^
235 parameters	Δρ_min_ = −1.05 e Å^−^^3^
0 restraints	

### Fractional atomic coordinates and isotropic or equivalent isotropic displacement parameters (Å^2^)

**Table:**

	*x*	*y*	*z*	*U*_iso_*/*U*_eq_	
C1	0.50019 (6)	0.26912 (11)	0.95475 (8)	0.0164	
C2	0.54654 (6)	0.20061 (11)	0.93269 (8)	0.0149	
C3	0.53732 (6)	0.08279 (11)	0.91462 (8)	0.0167	
C4	0.57355 (6)	0.02376 (11)	0.88215 (8)	0.0170	
C5	0.61874 (6)	0.08366 (11)	0.86547 (8)	0.0168	
C6	0.62966 (6)	0.20102 (11)	0.88333 (8)	0.0148	
C7	0.67218 (6)	0.27004 (11)	0.85493 (8)	0.0163	
C8	0.62887 (6)	0.33794 (11)	0.77464 (8)	0.0143	
C9	0.61781 (6)	0.45548 (11)	0.77855 (8)	0.0160	
C10	0.57288 (6)	0.51528 (11)	0.70662 (8)	0.0164	
C11	0.53667 (6)	0.45584 (11)	0.63009 (8)	0.0159	
C12	0.54589 (6)	0.33795 (11)	0.62379 (8)	0.0144	
C13	0.59403 (6)	0.28149 (10)	0.69587 (8)	0.0137	
C14	0.59478 (6)	0.25714 (10)	0.91977 (8)	0.0145	
C15	0.55982 (8)	−0.10064 (12)	0.85763 (10)	0.0239	
C16	0.56055 (8)	0.64034 (12)	0.71362 (10)	0.0231	
O17	0.60538 (4)	0.37378 (8)	0.93772 (6)	0.0152	
C18	0.65446 (7)	0.39656 (12)	1.02414 (8)	0.0203	
C19	0.66500 (8)	0.52464 (13)	1.03584 (10)	0.0267	
C20	0.71259 (8)	0.55535 (15)	1.12794 (11)	0.0327	
O21	0.60378 (5)	0.16480 (8)	0.69063 (6)	0.0160	
C22	0.65246 (7)	0.13892 (11)	0.66358 (9)	0.0186	
C23	0.64766 (7)	0.01154 (13)	0.64361 (10)	0.0246	
C24	0.69974 (8)	−0.02954 (15)	0.61997 (11)	0.0314	
Cl25	0.61811 (3)	−0.19788 (3)	0.93509 (3)	0.0381	
Cl26	0.62004 (3)	0.73262 (3)	0.70485 (3)	0.0407	
H11	0.5243	0.3212	1.0025	0.0157*	
H12	0.4747	0.2162	0.9728	0.0143*	
H31	0.5042	0.0438	0.9235	0.0150*	
H51	0.6414	0.0427	0.8393	0.0158*	
H72	0.7020	0.3210	0.9014	0.0138*	
H71	0.6988	0.2163	0.8411	0.0144*	
H91	0.6406	0.4933	0.8305	0.0136*	
H111	0.5048	0.4966	0.5830	0.0128*	
H151	0.5183	−0.1218	0.8522	0.0232*	
H152	0.5596	−0.1137	0.8038	0.0233*	
H162	0.5642	0.6536	0.7693	0.0219*	
H161	0.5193	0.6646	0.6685	0.0223*	
H181	0.6947	0.3556	1.0335	0.0207*	
H182	0.6413	0.3680	1.0656	0.0205*	
H192	0.6830	0.5531	0.9985	0.0289*	
H191	0.6222	0.5628	1.0206	0.0279*	
H201	0.7230	0.6366	1.1318	0.0433*	
H203	0.7532	0.5123	1.1473	0.0441*	
H202	0.6935	0.5370	1.1652	0.0450*	
H221	0.6956	0.1595	0.7082	0.0178*	
H222	0.6420	0.1815	0.6115	0.0173*	
H231	0.6530	−0.0287	0.6945	0.0258*	
H232	0.6051	−0.0045	0.5971	0.0253*	
H242	0.6953	−0.1109	0.6068	0.0429*	
H241	0.7421	−0.0116	0.6648	0.0429*	
H243	0.6957	0.0125	0.5702	0.0441*	

### Atomic displacement parameters (Å^2^)

**Table:**

	*U*^11^	*U*^22^	*U*^33^	*U*^12^	*U*^13^	*U*^23^
C1	0.0182 (6)	0.0177 (5)	0.0144 (5)	0.0010 (4)	0.0086 (5)	0.0009 (4)
C2	0.0162 (6)	0.0154 (5)	0.0129 (5)	0.0013 (4)	0.0066 (5)	0.0018 (4)
C3	0.0173 (6)	0.0153 (5)	0.0169 (6)	−0.0003 (4)	0.0076 (5)	0.0026 (4)
C4	0.0192 (6)	0.0138 (5)	0.0165 (5)	0.0009 (4)	0.0070 (5)	0.0011 (4)
C5	0.0185 (6)	0.0150 (5)	0.0169 (6)	0.0029 (4)	0.0084 (5)	0.0017 (4)
C6	0.0135 (5)	0.0160 (5)	0.0134 (5)	0.0012 (4)	0.0051 (4)	0.0027 (4)
C7	0.0144 (5)	0.0181 (5)	0.0160 (5)	0.0007 (4)	0.0066 (5)	0.0021 (4)
C8	0.0133 (5)	0.0147 (5)	0.0167 (5)	−0.0002 (4)	0.0085 (5)	0.0012 (4)
C9	0.0172 (6)	0.0152 (5)	0.0170 (6)	−0.0020 (4)	0.0092 (5)	−0.0009 (4)
C10	0.0194 (6)	0.0130 (5)	0.0200 (6)	−0.0006 (4)	0.0119 (5)	0.0006 (4)
C11	0.0166 (6)	0.0147 (5)	0.0172 (6)	0.0008 (4)	0.0086 (5)	0.0026 (4)
C12	0.0160 (6)	0.0151 (5)	0.0147 (5)	−0.0010 (4)	0.0092 (5)	0.0003 (4)
C13	0.0148 (5)	0.0125 (5)	0.0167 (5)	−0.0002 (4)	0.0098 (5)	0.0008 (4)
C14	0.0155 (6)	0.0131 (5)	0.0128 (5)	0.0004 (4)	0.0047 (4)	0.0012 (4)
C15	0.0292 (7)	0.0166 (6)	0.0248 (7)	−0.0011 (5)	0.0117 (6)	−0.0009 (5)
C16	0.0308 (7)	0.0146 (6)	0.0267 (7)	0.0017 (5)	0.0156 (6)	0.0007 (5)
O17	0.0169 (4)	0.0129 (4)	0.0131 (4)	−0.0005 (3)	0.0046 (3)	0.0000 (3)
C18	0.0218 (6)	0.0190 (6)	0.0142 (6)	0.0007 (5)	0.0035 (5)	−0.0003 (4)
C19	0.0296 (8)	0.0202 (6)	0.0237 (7)	−0.0023 (5)	0.0068 (6)	−0.0047 (5)
C20	0.0261 (8)	0.0328 (8)	0.0306 (8)	−0.0005 (6)	0.0059 (6)	−0.0149 (6)
O21	0.0188 (4)	0.0126 (4)	0.0208 (4)	0.0013 (3)	0.0127 (4)	−0.0002 (3)
C22	0.0197 (6)	0.0185 (6)	0.0220 (6)	0.0025 (5)	0.0134 (5)	−0.0003 (5)
C23	0.0223 (7)	0.0215 (6)	0.0300 (7)	0.0024 (5)	0.0122 (6)	−0.0073 (5)
C24	0.0253 (7)	0.0352 (8)	0.0327 (8)	0.0104 (6)	0.0126 (6)	−0.0073 (6)
Cl25	0.0615 (3)	0.01859 (16)	0.02742 (19)	0.01114 (16)	0.01501 (19)	0.00475 (13)
Cl26	0.0690 (3)	0.02000 (17)	0.0518 (3)	−0.01600 (18)	0.0438 (3)	−0.00762 (16)

### Geometric parameters (Å, °)

**Table:**

C1—C12^i^	1.5220 (17)	C14—O17	1.3837 (15)
C1—C2	1.5214 (18)	C15—Cl25	1.7998 (15)
C1—H11	0.973	C15—H151	0.950
C1—H12	0.998	C15—H152	0.958
C2—C3	1.3956 (17)	C16—Cl26	1.8037 (15)
C2—C14	1.3994 (17)	C16—H162	0.958
C3—C4	1.3927 (18)	C16—H161	0.957
C3—H31	0.962	O17—C18	1.4392 (15)
C4—C5	1.3940 (18)	C18—C19	1.503 (2)
C4—C15	1.4959 (19)	C18—H181	0.988
C5—C6	1.3922 (17)	C18—H182	0.970
C5—H51	0.967	C19—C20	1.521 (2)
C6—C7	1.5203 (18)	C19—H192	0.984
C6—C14	1.4018 (18)	C19—H191	1.000
C7—C8	1.5190 (17)	C20—H201	0.967
C7—H72	0.985	C20—H203	0.974
C7—H71	0.982	C20—H202	0.969
C8—C9	1.3933 (17)	O21—C22	1.4420 (16)
C8—C13	1.4016 (17)	C22—C23	1.5094 (19)
C9—C10	1.3938 (18)	C22—H221	0.971
C9—H91	0.927	C22—H222	0.969
C10—C11	1.3946 (18)	C23—C24	1.521 (2)
C10—C16	1.4929 (18)	C23—H231	0.966
C11—C12	1.3950 (17)	C23—H232	0.961
C11—H111	0.941	C24—H242	0.965
C12—C13	1.4024 (17)	C24—H241	0.952
C13—O21	1.3810 (14)	C24—H243	0.968
			
C12^i^—C1—C2	108.63 (10)	C4—C15—H151	110.3
C12^i^—C1—H11	109.8	Cl25—C15—H151	106.3
C2—C1—H11	110.8	C4—C15—H152	109.9
C12^i^—C1—H12	110.2	Cl25—C15—H152	108.1
C2—C1—H12	110.4	H151—C15—H152	108.5
H11—C1—H12	107.0	C10—C16—Cl26	112.72 (10)
C1—C2—C3	121.19 (11)	C10—C16—H162	108.1
C1—C2—C14	120.36 (11)	Cl26—C16—H162	106.6
C3—C2—C14	118.00 (12)	C10—C16—H161	111.8
C2—C3—C4	121.13 (12)	Cl26—C16—H161	105.1
C2—C3—H31	118.1	H162—C16—H161	112.5
C4—C3—H31	120.7	C14—O17—C18	112.95 (9)
C3—C4—C5	119.59 (12)	O17—C18—C19	108.92 (11)
C3—C4—C15	120.30 (12)	O17—C18—H181	107.9
C5—C4—C15	119.87 (12)	C19—C18—H181	111.8
C4—C5—C6	120.90 (12)	O17—C18—H182	111.4
C4—C5—H51	118.7	C19—C18—H182	108.7
C6—C5—H51	120.3	H181—C18—H182	108.1
C5—C6—C7	121.09 (12)	C18—C19—C20	111.62 (13)
C5—C6—C14	118.31 (12)	C18—C19—H192	109.3
C7—C6—C14	120.23 (11)	C20—C19—H192	108.3
C6—C7—C8	109.38 (10)	C18—C19—H191	108.8
C6—C7—H72	110.6	C20—C19—H191	108.6
C8—C7—H72	111.6	H192—C19—H191	110.1
C6—C7—H71	108.8	C19—C20—H201	109.7
C8—C7—H71	108.4	C19—C20—H203	110.9
H72—C7—H71	108.0	H201—C20—H203	107.8
C7—C8—C9	121.12 (11)	C19—C20—H202	109.9
C7—C8—C13	120.51 (11)	H201—C20—H202	110.0
C9—C8—C13	118.09 (11)	H203—C20—H202	108.5
C8—C9—C10	121.15 (12)	C13—O21—C22	113.71 (10)
C8—C9—H91	118.5	O21—C22—C23	107.23 (11)
C10—C9—H91	120.3	O21—C22—H221	110.3
C9—C10—C11	119.54 (11)	C23—C22—H221	111.5
C9—C10—C16	119.84 (12)	O21—C22—H222	108.5
C11—C10—C16	120.49 (12)	C23—C22—H222	108.5
C10—C11—C12	121.02 (12)	H221—C22—H222	110.7
C10—C11—H111	118.6	C22—C23—C24	112.83 (13)
C12—C11—H111	120.4	C22—C23—H231	106.9
C1^i^—C12—C11	121.15 (11)	C24—C23—H231	109.0
C1^i^—C12—C13	120.33 (11)	C22—C23—H232	108.9
C11—C12—C13	118.09 (11)	C24—C23—H232	109.3
C12—C13—C8	121.91 (11)	H231—C23—H232	109.8
C12—C13—O21	118.79 (11)	C23—C24—H242	111.2
C8—C13—O21	119.11 (11)	C23—C24—H241	110.1
C6—C14—C2	121.86 (11)	H242—C24—H241	111.5
C6—C14—O17	118.64 (11)	C23—C24—H243	109.5
C2—C14—O17	119.34 (11)	H242—C24—H243	108.6
C4—C15—Cl25	113.61 (10)	H241—C24—H243	105.7

Symmetry codes: (i) −*x*+1, *y*, −*z*+3/2.

### Hydrogen-bond geometry (Å, °)

**Table:**

*D*—H···*A*	*D*—H	H···*A*	*D*···*A*	*D*—H···*A*
C22—H222···Cl25^ii^	0.97	2.90	3.786 (1)	153
C23—H231···Cl26^iii^	0.97	2.90	3.557 (2)	127

Symmetry codes: (ii) *x*, −*y*, *z*−1/2; (iii) *x*, *y*−1, *z*.
